# GFAP-Cre-Mediated Transgenic Activation of Bmi1 Results in Pituitary Tumors

**DOI:** 10.1371/journal.pone.0035943

**Published:** 2012-05-04

**Authors:** Bart A. Westerman, Marleen Blom, Ellen Tanger, Martin van der Valk, Ji-Ying Song, Marije van Santen, Jules Gadiot, Paulien Cornelissen-Steijger, John Zevenhoven, Haydn M. Prosser, Anthony Uren, Eleonora Aronica, Maarten van Lohuizen

**Affiliations:** 1 Division of Molecular Genetics, The Netherlands Cancer Institute, Amsterdam, The Netherlands; 2 Department of Oncogenomics, Academic Medical Center, Amsterdam, The Netherlands; 3 Department of Animal-Pathology, The Netherlands Cancer Institute, Amsterdam, The Netherlands; 4 Department of Pathology, Academic Medical Center, Amsterdam, The Netherlands; 5 The Wellcome Trust Sanger Institute, Wellcome Trust Genome Campus, Hinxton, Cambs, United Kingdom; 6 Cancer Genomics group at the MRC Clinical Sciences Centre, London, United Kingdom; 7 Department of Neuro-Pathology, Academic Medical Center, Amsterdam, The Netherlands; 8 The Centre of Biomedical Genetics, Academic Medical Center, Amsterdam, The Netherlands; University of Florida, United States of America

## Abstract

Bmi1 is a member of the polycomb repressive complex 1 and plays different roles during embryonic development, depending on the developmental context. Bmi1 over expression is observed in many types of cancer, including tumors of astroglial and neural origin. Although genetic depletion of Bmi1 has been described to result in tumor inhibitory effects partly through INK4A/Arf mediated senescence and apoptosis and also through INK4A/Arf independent effects, it has not been proven that Bmi1 can be causally involved in the formation of these tumors. To see whether this is the case, we developed two conditional Bmi1 transgenic models that were crossed with GFAP-Cre mice to activate transgenic expression in neural and glial lineages. We show here that these mice generate intermediate and anterior lobe pituitary tumors that are positive for ACTH and beta-endorphin. Combined transgenic expression of Bmi1 together with conditional loss of Rb resulted in pituitary tumors but was insufficient to induce medulloblastoma therefore indicating that the oncogenic function of Bmi1 depends on regulation of p16^INK4A^/Rb rather than on regulation of p19^ARF^/p53. Human pituitary adenomas show Bmi1 overexpression in over 50% of the cases, which indicates that Bmi1 could be causally involved in formation of these tumors similarly as in our mouse model.

## Introduction

Bmi1 is part of the polycomb repressive complex 1 (PRC1), a transcriptional repressive complex that silences genes during embryonic development. PRC1 complexes act through recognition of H3K27Me3 epigenetic tags on histone 3 [1], [2] and through ubiquitin ligase activity towards K119 on histone 2 [3], [4].

Among the Bmi1 target genes are regulators of stem cell self-renewal, partially by repressing the senescence and apoptosis-regulating genes p16**^INK4A^** and p19**^Arf^** [5]. In mice, loss of Bmi1 results in hematopoietic and neural defects leading to an early death (mostly within 8 weeks). The partial rescue of Bmi1 deletion by simultaneous deletion of p16**^INK4A^** and p19**^Arf^** suggested that p16**^INK4A^**/p19**^Arf^** independent targets are responsible for the severe phenotype in mice [6], [7]. It was shown that Chk2 deletion was able to substantially rescue this severe phenotype implicating mitochondrial function and redox homeostasis as target processes of Bmi1 [8]. Furthermore, Bmi1 has been shown to repress p21 in the cerebellum [9] and in the developing forebrain. This latter repression is mediated through FoxG1 [10], [11]. Polycomb proteins also affect developmental competence of neural precursors during glial and neural differentiation [12].

In cancer, both p16**^INK4A^** and p19**^Arf^** dependent as well as independent oncogenic functions of Bmi1 have been shown [7], [13]. The mechanism behind this might be diverse and context dependent. PRC1 plays a role in DNA double strand break repair [14]. Furthermore, Bmi1 is required to prevent glial differentiation in SmoM2 induced medulloblastoma [15], possibly by a similar mechanism of Ezh2 mediated repression of BMPR1A that affects JAK/STAT3 mediated glial differentiation in glioma [16]. In addition, Bmi1 was shown to regulate Twist1 mediated epithelial-mesenchymal transition [17].

Many reports have shown over expression of Bmi1 in tumors of astroglial, neural or neuroendocrine origin ([18]; summary in **Table 1**). Therefore, it is assumed that the proto-oncogene Bmi1 positively contributes to tumor formation in these cases. However, a causal role for Bmi1 in the generation of these tumors has not been demonstrated [19], [20]. We show here that Glial fibrillary acidic protein-Cre (GFAP-Cre) mediated conditional over expression of Bmi1 generates anterior and intermediate lobe pituitary tumors. In addition, we show that Bmi1 over expression cannot substitute for p53 loss in a predisposing genetic model of medulloblastoma which indicates that the oncogenic function of Bmi1 is mediated by p16**^INK4A^**/Rb mediated effects rather then through p19**^ARF^**/p53 effects.

**Table 1:** Reported over expression of Bmi1 in astroglial and neural tumors.

	Tumor	Reference
Central & peripheral	Astrocytoma	[33]
nervous system tumors	Ependymoma	[18]
	Glioma	[13]
		[19]
	Oligodendroglial tumours	[34]
		[18]
	Medulloblastoma	[35]
		[36]
	Meningoma	[18]
	Neuroblastoma	[37]

## Materials and Methods

### Transgene Model

All animal experiments have been conducted with approval of the ethical committee of the Netherlands Cancer Institute under references 04.003 B21/04.003 B38ext and 04.003 B23/04.003 B39ext. Genetically engineered mice with predispositions leading to cancer were checked twice every week for the occurrence of tumors. Animals were immediately sacrificed when a tumor was detected. All procedures have been conducted according to the standard operating procedures of the institute.

**Figure 1:**
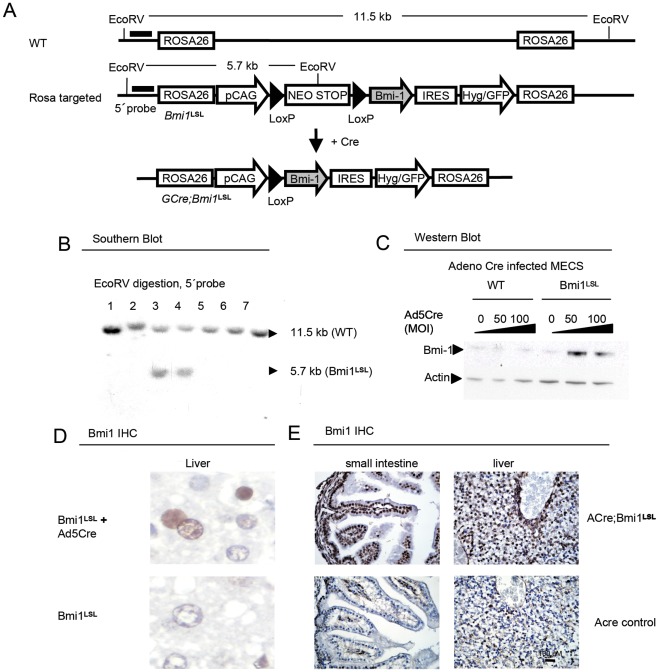
Bmi1^LSL^ conditional transgenic mice express Bmi1 after Cre mediated activation. (A) Strategy for targeting the CAG promotor-LoxP-PGK-Neo-3xtranscriptionstop-LoxP-Bmi1 cDNA-IRES-Hyg/eGFP cassette into the ROSA26 locus. (B) Southern blot showing germ line transmission of two out of 7 mice. (C) Western blot showing Adenoviral-Cre mediated expression of Bmi1 in cultured mammary epithelial cells (MECS) of a Bmi1**^LSL^** transgene mouse compared to a wild type (WT) mouse. Bmi1 is detected in MECS that received 50 or 100 virus particles per cell. MOI, multiplicity of infection. (D) Immunohistochemistry showing nuclear transgenic Bmi1 expression of hepatocytes of adult mice after adenocre (Ad5Cre) mediated activation using intravenous injection of 10^9^ infectious particles. (E) Immunohistochemistry showing Bmi1 expression in the small intestine and liver of Actin-Cre (Acre) or Acre;Bmi1 mice of embryonic day 18.5 mice.

The cDNA encoding mouse Bmi1 with a downstream internal ribosome entry site (IRES)-eGFP cassette was cloned in the Rosa 26 targeting construct CEB11. This construct contains three transcriptional stop (poly A) signals flanked by LoxP sites to enable removal of this stop cassette upon LoxP-recombination (**Figure 1A**). The construct was targeted to the Rosa26 locus by homologous recombination which was confirmed by Southern blot analysis (**Figure 1A, B**). In addition, we generated an independent conditional targeting construct with mutant 66/71 LoxP-recombinase sites enabling reversal of the antisense cloned Bmi1 cDNA (**Figure S1A, C** and results not shown). The first mouse model is referred to as Bmi1**^LSL^** and the latter as Bmi1**^Lox66/Lox71^**. Detailed information on the transgenes is available at MGI (Mouse Genome Informatics database, http://www.informatics.jax.org/). Reference numbers are: Bmi1**^LSL^**, MGI:4398910, Gt(ROSA)26Sor<tm1(CMV-Bmi1,-EGFP)Nki>, also named Bmi-CTS, when inbred on a FVB background: NKI strain# 1353, when inbred on a C57BL/6 background: NKI strain# 1656. Bmi1**^Lox66/Lox71^**, MGI:4398914, Gt(ROSA)26Sor<tm2(CMV-Bmi1,EGFP)Nki>, also named Bmi-CTI, when inbred on a FVB background: NKI strain# 1148, when inbred on a C57BL/6 background: NKI strain# 1657.

The Bmi1**^LSL^** transgenic strain was subsequently crossed with Actin-Cre (Acre) strain to analyze the phenotype when Bmi1 is activated constitutively throughout the mouse. In addition, transgenic mice were crossed to Glial fibrillary acidic protein promoter driven Cre (GFAP-Cre or GCre, (MGI#2663939; Tg(Gfap-cre)2Brn), [21]) to conditionally activate Bmi1 expression in GFAP expressing cells. Mice carrying conditional mutant forms of Rb (Rb**^LoxP^**, [21]) were crossed with the GCre; Bmi1**^LSL^** to analyze the contribution of Bmi1 over expression on top of this background. All breeding was done on an FVB background. All tumors were analyzed by the pathologists of the NKI and in case of doubt, dr. Annemiek Rosemöller, of the VU Medical Center, Amsterdam was consulted.

### Intravenous Injection of Adeno-cre Virus

As a positive control for transgenic expression, we induced expression of Bmi1 from the Bmi1**^LSL^** locus by LoxP recombination in the liver by intravenous injecting adenovirus which expressed Cre (Ad5-Cre, obtained from the Gene transfer Vector Core, University of Iowa). For this, 2×10^9^ virus particles per mouse (estimated 2% infection of hepatocytes) in a total volume of 100 µl was injected. Wild type mice were treated simultaneously as a reference. One week before the experiment, the mice were given cyclosporine (Novartis Neoral, 0.1 mg/ml in acidified water) to reduce immunosupression of adenovirally infected cells. Rosa reporter mice (R26R) which have a Cre inducible beta galactosidase gene targeted to the Rosa26 locus were used as a control for the viral infection (based on [22]).

### Immunohistochemistry, Electron Microscopy and FACS Analysis

For immunohistochemistry, tissue was fixed overnight in 10% neutral buffered formalin. Microwave antigen retrieval was performed by boiling in sodium citrate buffer for 20 minutes. The following primary antibodies were used: Bmi1 (mouse monoclonal clone F6, Upstate, 1∶50 (in normal tissue and human tumors) or 1∶200 (in mouse tumors unless otherwise stated)), ACTH (Organon 11150), hGH (DAKO A570), LH (C93, DAKO M3502), Prolactine (DAKO A569), TSH (DAKO A574), NCAM-1 (123C3.D5, Neomarkers MS-204), CAM 5.2 (B&D 349205), Chormogranine A (DAKO A430), anti-human Ki 67 (MIB-1, DAKO M7240), anti-mouse Ki 67 (TEC3, DAKO M7249), Phospho-Histone H3 (Upstate 06-570), glial fibrillary acidic protein (GFAP, Biotrend; 4650–0100; 1∶10), S-100 protein (S-100, DakoCytomation: Z0311; 1∶2000 ), synaptophysin (SYN, DakoCyomation; A0010; 1∶100), p75 NTR, also named low affinity nerve growth factor (NGF) receptor (p-75, Chemicon; AB1554; 1∶8000), F4/80 antigen, a 160 kD glycoprotein expressed by murine macrophages (F4/80, Serotec; MCAP497; 1∶400), neurofilament (NF, Biotrend; NA1297; 1∶2000), Keratin-8 (University of Iowa; Troma1; 1∶600). Antibodies were detected by peroxidase staining using the Powervision system (Immunologics) followed by visualization on a Zeiss Axiovert microscope.

For electron microscopy, a selected area from paraffin-embedded material was dissolved in 1% osmiumtetroxide in toluene and embedded in epoxyresin LX-112. Light microscopy sections were stained with toluidine blue. EM sections were stained with tannic acid, uranyl acetate and lead citrate and examined in a Philips CM10 transmission electron microscope (FEI, Europe BV, Eindhoven, the Netherlands).

Detection of eGFP in recombined transgenic ES cells was done on a Facs scan (BD).

## Results

### Bmi1 is Over Expressed Efficiently in Two Conditional Transgenic Mouse Strains

Bmi1 is a proto-oncogene which was identified by its ability to initiate lymphoid tumors [23]. To analyze whether Bmi1 is a *bona fide* oncogene in non-lymphoid compartments, we analyzed the effects of over expression of Bmi1 in mice. For this, we developed a conditional lox-stop-lox Bmi1 transgene model, further on referred to as Bmi1**^LSL^**. In this model, Bmi1 is conditionally over expressed from the ROSA26 locus, under control of a combined CMV and beta-Actin (CAG) promoter. This enables constitutive expression of Bmi1 in the cellular compartment of interest independent of the transcriptional signals that normally regulate Bmi1 (**Figure 1A**). We simultaneously used an alternative transgene strategy, where the Bmi1 encoding cDNA was cloned in an inverted position downstream of the CAG promoter. This inverted cDNA can be reverted in a sense orientation by using Cre mediated recombination of the LoxP66 and LoxP71 sites that flank the cDNA (shown in **Figure S1A)**. This second mouse, referred to as Bmi1**^Lox66/Lox71^**, showed identical characteristics as the Bmi1**^LSL^** targeted mouse (**Figure S1C** and results not shown). All subsequent steps described below were performed with the Bmi1**^LSL^** mice.

Proper gene targeting was confirmed by Southern blot (**Figure 1B**). Targeted ES cells and in vitro cultured mammary epithelial cells (MECs) of transgenic mice showed Cre-mediated recombination as seen from IRES-eGFP mediated expression and over expression of Bmi1, respectively (**Figure S1B,** **Figure 1C**). In addition, intravenous injection of adenovirus encoding the Cre recombinase in adult transgenic mice resulted in over expression of Bmi1 in adult hepatocytes, which normally lack Bmi1 expression (**Figure 1D**).

### Constitutive Over Expression of Bmi1 is Neonatally Lethal

**Figure 2:**
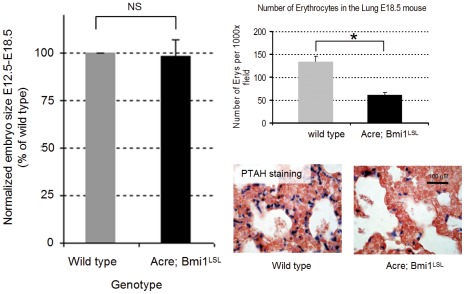
Constitutive transgenic expression of Bmi1^LSL^ is neonatally lethal. (A) Histogram showing that constitutive expressing Acre;Bmi1**^LSL^** mice have the same growth kinetics as wild type control mice in utero between embryonic day E12.5 and E18.5. (B) Histogram showing that Acre;Bmi1**^LSL^** transgene mice have less nucleated red blood cells in their lungs compared to Acre control mice at embryonic day E18.5. Nucleated red blood cells are visualized using PTAH staining resulting in blue nuclei, as shown in the lower panels. *p = <0.05, NS: not significant.

To analyze the global effect of constitutive Bmi1 expression in mice, we generated Actin-cre (Acre); Bmi1**^LSL^** mice. These animals have transgenic over expression during embryogenesis, as is shown by over expression of Bmi1 in cell types that normally lack or have low endogenous expression levels such as small intestine and liver at embryonic day 18.5 (**Figure 1E**). We never observed living Acre;Bmi1**^LSL^** pups (none out of 23 neonatally genotyped mice was Acre;Bmi1**^LSL^** positive, **Table 2**). In contrast, in utero, we observed genetic inheritance of the transgenic allele in mendelian ratios (7 Acre;Bmi1**^LSL^** positives out of 38 mice (18%) with an expected frequency of 25%, p>0.1) and mice that were alive were observed until day E15.5-E18.5 suggesting that a neonatal defect is the reason for the death of the mice. Since Bmi1 deficient mice have strong growth defects, we analyzed growth during embryonic development (**Figure 2A**). From this we did not observe any obvious growth effects compared to matched wild type littermates. Additional analysis of the heart and brain did not show any obvious defects. We also looked for hematological defects since polycomb proteins are regulators of normal hematopoietic development [24], [25]. Although no gross hematological defects were detected, we found that transgenic mice had a significant reduction (over 50%) of nucleated red blood cells in the lungs at embryonic day 18.5, as shown by PTAH staining [26], **Figure 2B**). Together these results show that Acre;Bmi1**^LSL^** transgenic mice express high levels of Bmi1 which results in a neonatally lethal phenotype.

**Table 2:** Mendelian distribution of transgenic allele before and after birth.

Genotype	Stage	Double transgene	Total	Mendelian ratio	Expected ratio
Acre;Bmi1	E12.5–E18.5	7	38	18%	25%
Acre;Bmi1	Neonatal	0	16	0%	25%

### Transgenic Expression of Bmi1 Induces Pituitary Tumors

**Figure 3:**
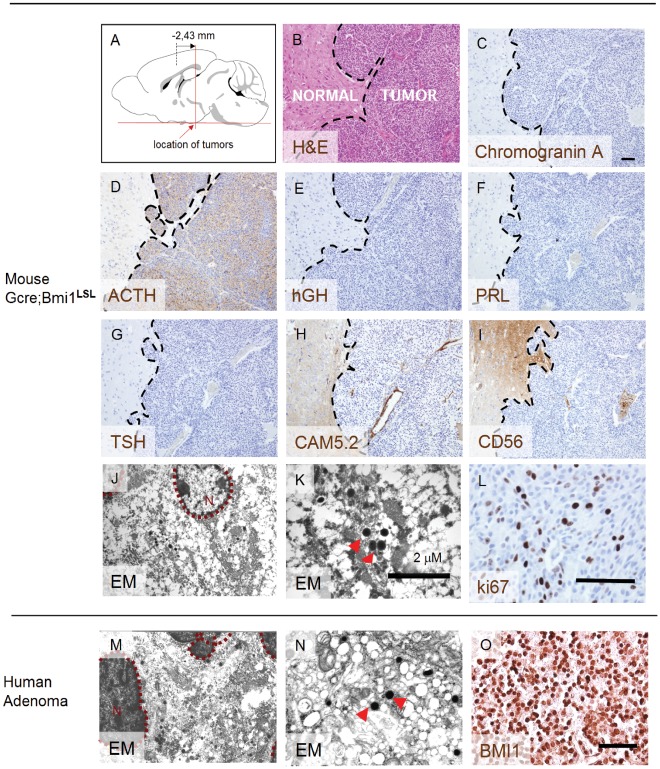
Histology of a representative example of a typical pituitary tumor as found in this study. (A) Average location of pituitary tumors observed in the GCre;Bmi1^LSL^ mice showing localization to the pituitary gland (n = 6). Localization is based on Allen’s mouse brain database, (B) H&E staining of a coronal section of the brain showing a typical pituitary tumor. Immunohistochemistry shows expression analysis of (C) Chromogranin A, (D) adrenocorticotropic hormone ACTH, (E) Growth Hormone hGH, (F) Prolactin PRL, (G) Thyroid Stimulating Hormone TSH, (H) cytokeratin 8/18 CAM5.2 and (I) CD56/NCAM. Of these markers, ACTH is clearly positive in this tumor. Electron microscopy (EM) shows secretory vesicles (indicated by the arrowheads) in Bmi1 transgenic tumors (J, K) in a similar way as observed in human tumors (M, N). Ki67 is clearly positive in this tumor (L). Immunohistochemistry of human pituitary adenomas show over expression of BMI1, which is visualized by a nuclear signal (O). Bars, 100 µm unless otherwise noted.

**Figure 4:**
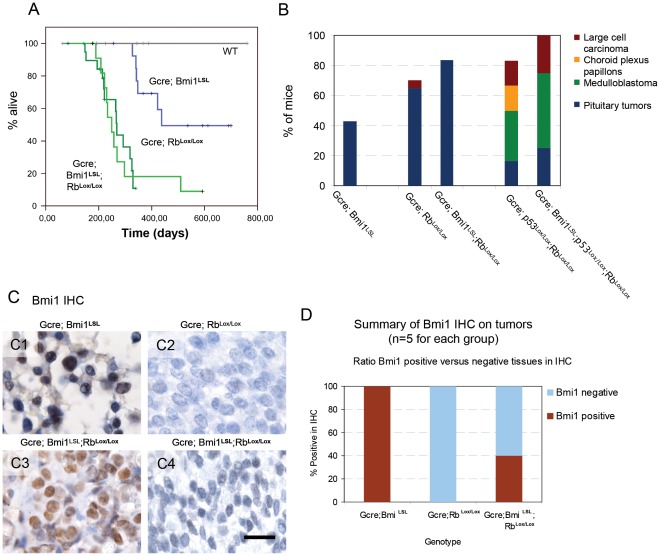
Transgenic expression of Bmi1 is sufficient to induce intermediate and anterior lobe pituitary tumors but does not induce medulloblastoma or glioma. (A) Kaplan Meier survival curves of mice carrying tumors because of GCre induced transgenic expression of Bmi1 complemented with loss of Rb. These data show that Bmi1 transgenic mice develop pituitary tumors after about one year. Pituitary tumors are also observed in Rb^Lox/Lox^ and Bmi1^LSL^;Rb^Lox/Lox^ transgenic mice. (B) Histograms showing the relative frequency and penetrance of tumors generated by the individual genotypic groups. All genotypes shown are GCre positive. Total cohort size: GCre;Bmi1^LSL^ n = 14, GCre;Rb^Lox/Lox^ n = 20, GCre;Rb^Lox/Lox^;Bmi1^LSL^ n = 12, GCre;p53^Lox/Lox^;Rb^Lox/Lox^ n = 6, GCre;p53^Lox/Lox^;Rb^Lox/Lox^; Bmi1^LSL^ n = 4, WT mice did not develop tumors, n = 7. (C) IHC results showing transgenic Bm1 expression in tumors raised on a GCre;Bmi1^LSL^ background (5/5), no expression in tumors raised on a Gcre;Rb^lox/lox^ background (0/5) while some of the GCre;Bmi1^LSL^; Rb^lox/lox^ mice were positive (2/5, 40%). These results are summarized in a histogram (D). Bar is 50 µm.

A causal role for Bmi1 in the generation of astroglial or neural tumors was not observed in earlier studies. We crossed Glial fibrillary acidic protein promoter driven Cre recombinase (GFAP-Cre) mice [21] with the Bmi1**^LSL^** mice allowing transgenic expression of Bmi1 in GFAP positive cells, which encompass different types of mature astrocytes as well as neural progenitors. Interestingly, these mice generated tumors with a latency of about one year, which shows that transgenic over expression of Bmi1 is sufficient to generate solid tumors (**Figure 3**, upper left panel). Out of the 16 GFAP-Cre;Bmi1**^LSL^** transgenic mice, 8 developed tumors (50%) of which 6 tumors showed intracranial localization. In addition, a mammary tumor and a primitive neuroectodermal tumor (PNET) were found. The intracranial tumors consisted of cells of uniform size with round to oval nuclei with a fine chromatin patterning and a moderate to abundant amount of pale cytoplasm. These tumors are referred to as typical and this was observed in 5 out of 6 cases. In one case we observed an atypical form with anaplastic cells with a pleiomorphic nuclei and a thin rim of pale cytoplasm. This latter form was sometimes observed in combination with the typical form as well. The highly vascularised tumors localized to the pituitary gland/hypothalamus as observed from their caudal localization relative to the brain (**Figure 3A**, average at -2,43 ± 0.475 mm bregma (based on coordinates of the Allen’s mouse brain database, mouse.brain-map.org)). To show that the tumors are a result of transgenic over expression of Bmi1, we stained -non transformed- postnatally derived (day 8) pituitary glands of Gcre;Bmi1**^LSL^** mice, which showed enhanced expression when compared to the wild type control (**Figure S2**). To confirm that the tumors were derived from endocrine cells of the pituitary gland, the tumors were subjected to immunohistochemistry using a panel of pituitary hormones. This showed that the typical tumors were positive for adrenocorticotropic hormone (ACTH, **Figure 3B–G** **, summarized in** **Table 3**). Additional electron microscopy analysis showed granular secretory vesicles (**Figure 3** **J and K**) as are commonly observed in hormone producing pituitary adenomas (**Figure 3** **M and N**).The number of Ki67 positive cells (**Figure 3** **L, Figure S4**) ranged from less than 1% to up to 8.4% comparable to human pituitary specimens that typically show between 1% to 3.8% positivity (MIB index). Cytokeratin 8/18 as stained by the CAM5.2 antibody was negative (**Figure 3H** and results not shown, respectively). Transgenic Bmi1 expression was observed in the tumors, as shown by the nuclear staining (**Figure 4C** **1**). Additional analysis of neuroendocrine markers (**Figure S3, summarized in Table S1**) showed that the tumors stained positive for the intermediate lobe pituitary marker beta-endorphin (B-END) and synaptophysin (SYN), which are commonly observed in pituitary adenomas [27]. No neuronal differentiation was observed both morphologically as well as indicated by absence of the neuronal marker NF. In addition, the tumors did not show expression of the astroglial markers GFAP and S-100 and were negative for the glial/neural progenitor and hemo-fibrous marker p75**^NGFR^** (not shown) and the glandular epithelial marker KER8. The tumor that showed the atypical cytomorphology had a different marker profile consisting of a mosaic pattern of expression of the neuroendocrine markers (see **Table 3**). Together, our results show that the majority of the tumors in the transgenic mice resemble anterior lobe neuroendocrine cells of the pituitary gland.

**Table 3:** IHC analysis of pituitary hormones in Gcre; Bm1^LSL^ induced tumors.

Marker	Typical tumor (n = 5)	Atypical tumor (n = 1)
GH	**–**	Mosaic
PRL	**–**	Mosaic
ACTH	**+**	Mosaic
TSH	**–**	Mosaic
FSH/LH	**n.d.**	**n.d.**
NULL	**n.o.**	**n.o.**

n.d., not done, n.o., not observed.

Human pituitary adenomas are derived from the anterior lobe [28]. To analyze whether BMI1 is over expressed in human pituitary adenomas, we performed immunohistochemistry. For this, 13 clinical specimens representing non functioning and secretory pituitary tumors were analyzed. This showed that 7 out of 13 (54%) of the specimens analyzed showed over expression of BMI1 of which 42% was considered strongly positive as compared to control tissues including normal brain. A representative case is shown in **Figure 3O**. These results show that a significant portion of human pituitary adenomas has over expression of BMI1.

### Analysis of Transgenic Expression of Bmi1 in a Predisposing Background for Medulloblastoma

Combined loss of p53 and Rb mediated by GFAP-Cre in the cerebellum leads to medulloblastoma [21] and this was shown to be caused by activation of the Shh pathway mediated by loss of Ptc [29]. Bmi1 is necessary for the progression of Shh induced medulloblastoma [15]. Since Bmi1**^LSL^** potentially represses p19**^ARF^**/p53 function, we anticipated that GFAP-Cre; Bmi1**^LSL^**; Rb**^Lox/Lox^** mice might form medulloblastomas. To test this hypothesis, we crossed GFAP-Cre;Bmi1**^LSL^** on a Rb**^Lox/Lox^** background (n = 12). We analyzed the incidence of tumors and these were histologically classified by two independent pathologists (**Figure 4A and B**). From this breeding we did not observe medulloblastoma upon transgenic over expression of Bmi1 and instead virtually all tumors observed in the GFAP-Cre;Bmi1**^LSL^**; Rb**^Lox/Lox^** mice were pituitary tumors (**Figure 4B**). As a control, we used mice that were deficient for both Rb and p53 (GFAP-Cre; p53**^Lox/Lox^**; Rb**^Lox/Lox^**, n = 6) and these formed medulloblastomas with an early onset as expected (**Figure 4B** and results not shown). Addition of transgenic expression of Bmi1 transgene resulted in a similar amount and penetrance of medulloblastomas (GCre;p53**^Lox/Lox^**;Rb**^Lox/Lox^;** Bmi1**^LSL^,** n = 4). These data indicate that the oncogenic function of Bmi1 in GFAP positive cells depends on p16**^INK4A^**/Rb regulation and less on p19**^ARF^**/p53 regulation.

Earlier reports describing pituitary tumors because of Rb loss described a similar localization and histology [30], [31], [32], and we tested whether Bmi1 overexpression has an enhanced role on top of Rb loss (GCre;Rb**^Lox/Lox^**;Bmi1**^LSL^** n = 12, GCre;Rb**^Lox/Lox^** n = 20). Although we did observe transgenic Bmi1 expression (**Figure 4C**) in these mice (in two out of five mice, see histogram in **Figure 4D**), no enhanced incidence of pituitary tumors was observed when compared to mice that were Rb deficient. Since the oncogenic function Bmi1 is dependent on the p16**^INK4A^**/Rb pathway, deletion of the locus by recombination (which, based on the lack Bmi1 overexpression, happened in 60% of the cases) is therefore dominant over inactivation the locus by Bmi1 over expression (which happens in in 40% of the cases).

Together, our results show that transgenic over expression of Bmi1 is sufficient to generate adrenocorticotropic pituitary tumors and a subset of clinical specimens of pituitary adenomas show high expression of Bmi1. The phenotype of Bmi1 over expression overlaps with the phenotype observed in Rb deficient mice and absence of medulloblastoma in GFAP-Cre; Bmi1**^LSL^**; Rb^L**ox/Lox**^ mice, shows that the transgenic Bmi1 over expression is insufficient to functionally inactivate the p19**^ARF^**/p53 pathway and indicates that the oncogenic role of Bmi1 is primarily dependent on repression of p16**^INK4A^**/Rb.

## Discussion

Bmi1 is a proto oncogene and it’s over expression has been observed in many tumors of neural and astroglial origin (**Table 1**). Amplification of Bmi1 has been seen in 11% of mantle cell lymphomas [38] and chromosomal gains have been seen in high-grade astrocytomas and ovarian cancer [39], [40]. Other human neoplasms, including colon, breast and Laryngeal squamous cell carcinoma show no amplification of Bmi1 although frequent protein over expression is observed in these tumors [38]. Over expression by non-genetic causes is likely to result from convergence from multiple inputs acting on the transcriptional and posttranscriptional level [41], [42], [43], [44], [37]. By using a transgenic over expression model, we show that GFAP-Cre mediated transgenic over expression of Bmi1 is sufficient to drive the formation of pituitary tumors, a type of tumor that represents 10 to 25% of all intracranial neoplasms in humans.

The incomplete penetrance and long latency period (1 year) of tumor formation upon Bmi1 over expression suggested that additional genetic or epigenetic defects might facilitate the generation of tumors. Loss of Rb itself has been shown to induce pituitary tumors in mouse models [30], [31], [32] and loss of Rb or p16**^INK4A^** expression by hypermethylation is a common mechanism in human pituitary tumors [46], [47], [48]. We found a higher penetrance of pituitary tumors in the Rb deficient mice than in the Bmi1 transgene model which indicates that transgenic Bmi1 expression acts as a predisposing condition facilitating the silencing of the p16**^INK4A^** locus or, alternatively, points to an incomplete transcriptional repression of the p16**^INK4A^** pathway or could simply reflect the efficiency of Cre-mediated recombination events for the alleles used. Since no higher incidence of pituitary tumors was found upon combined Rb loss and Bmi1 over expression, this indicates that the oncogenic function of Bmi1 is dependent on the presence of a functional p16^INK4A^ locus, this is substantiated by the observation that in the latter case, 40% of the tumors are positive in IHC for Bmi1, indicating that the remaining 60% of the tumors have inactivated the Rb allele independent of Bmi1 over expression.

We do not know at which stage GFAP-Cre mediated transgene activation is accomplished in our transgenic model although enhanced expression of Bmi1 in Gcre;Bmi1**^LSL^** mice in non transformed cells of the pituitary gland of postnatal day 8 mice indicates that that transgenic activation has occured before this time point. The occurrence of pituitary tumors using GFAP-Cre mediated recombination of Rb has been shown before [29]. GFAP is expressed at different stages during development, which includes astrocytes of the sub ventricular zone [49] and in radial glial cells of the outer sub ventricular zone [50] both of which can act as a source of neural stem cells. GFAP expression can be activated by tumor stem cells of pituitary adenomas [51]. In this context it is interesting to note that transgenic mice generated from a Nestin-Bmi-1-GFP cassette did not generate tumors [19]. Neural stem cells isolated from these mice showed significant *in vitro* effects, however, *in vivo* effects were moderate, reflecting the fact that high activity of p16**^INK4^** and p19**^ARF^** are seen in cultures of neural stem cells, thereby increasing the reliance of cells upon Bmi1. In contrast, p16**^INK4^** and p19**^ARF^** are not detectably expressed by neural stem/progenitor cells in developing or young adult mice [52], [53] and therefore these neural stem cells are none or weakly responsive to elevated levels of Bmi1. Since we generated over expression of Bmi1 in GFAP expressing cells by using a Lox recombination system, we did not have a transient expression restricted to stem cell compartment but rather a constitutive over expression in all progeny of GFAP positive cells throughout later life, thereby enabling to reveal the oncogenic role of Bmi1.

We show that the tumors in the transgenic mice were localized to the pituitary gland, although this could be shown for large intracranial tumors only because of lack material of earlier neoplastic stages. The majority (n = 5 out of 6) of the tumors showed a typical cytomorphology and these tumors expressed ACTH. One tumor expressed multiple markers in a mosaic pattern and was referred to as an atypical type (**Table 3**). Neuroendocrine ACTH producing cells normally comprise 10–30% of the pituitary gland and 10% of pituitary tumors are positive for ACTH [54], indicating that our model enhances or selects tumorigenesis in ACTH positive cells. Previously, ACTH expression in pituitary tumors has been genetically linked to Rb function [55], [56] therefore indicating that our model selects for or enhances tumor formation in a similar population of cells as cells that have lost Rb function as discussed above.

Transgenic expression of Bmi1 failed to generate medulloblastoma even in the presence of the predisposing deletion of Rb. In contrast, the p53/Rb double deficient mice control mice developed medulloblastoma after half a year as expected [21], [29] thereby implicating that Bmi1 over expression is insufficient to fully functionally repress p19**^ARF^**/p53. Hence, our data point to a Rb mediated function of Bmi1 in our model. The p16^INK4A^ or the p53 pathways are frequently mutated in glioblastoma [57], [58], [59], however we have currently no indication that transgenic over expression of Bmi1 contributes to the development of glioma/glioblastoma, although other or additional predisposing lesions might enable this.

In our mouse model, we identified both anterior and intermediate lobe tumors. In contrast, human pituitary adenomas are considered to be derived from the anterior lobe only since all tumors resemble cell types of the anterior lobe hormone producing cell types [60]. Human pituitary adenomas show expression of BMI1 in 54% (our data) to up to 100% [34]. Furthermore, the pituitary gland might be primed to enable BMI1 to exert its oncogenic functions because the normal adult pituitary gland expresses all required PRC1 components RING1, MEL18, HPH1, RYBP [34]. These data show that over expression of Bmi1 does not only induce the generation of tumors in mice but this over expression is also commonly seen in established human pituitary adenomas and BMI1 could therefore be considered as a potential drug target for pituitary tumors.

In conclusion, we show here that Bmi1 transgene over expression is sufficient to drive pituitary tumors and we also show that more than 50% of clinical pituitary adenomas have high expression of Bmi1. Furthermore, Bmi1 initiated tumorigenesis is not enhanced by Rb loss which shows that the oncogenic function is largely dependent on Rb function and not on p53 function, which is further substantiated by the observation that Bmi1 overexpression is not sufficient to repress p53 sufficiently to generate medulloblastomas.

## Supporting Information

Figure S1 (A) Comparison of the two targeting constructs for conditional over expression of Bmi1. Two LoxP recombination methods are used, the Bmi1**^LSL^** construct contains a transcriptional stop sequence that can be removed by LoxP recombination and the Bmi1**^Lox66/Lox71^** construct contains two partially mutated LoxP sites that recombine upon Cre expression resulting reversion of the DNA that is flanked by the LoxP sites which results in the formation of one recombined LoxP site that has a low chance of reversal of the recombination process. ES cells that were targeted with the Bmi1**^LSL^** construct (B) or Bmi1**^LOX66/LOX71^** construct (C) show eGFP expression after Cre mediated activation of the transgene.
(TIF)

Figure S2 **Bmi1 immunohistochemistry of postnatal day 8 pituitary glands (upper panel) shows enhanced expression of Bmi1 in Gcre;Bmi1^LSL^ mice (middle panel) when compared to a wild type controls (lower panel).** The Bmi1 antibody was used at a concentration of 1∶50. Bar is 10 µm.
(TIF)

Figure S3 **Immunohistochemistry shows that Bmi1 transgenic mice generate pituitary tumors that stain positive for (A) beta-endorphin (B-END) and (B) synaptophysin (SYN).** The tumor is negative for (C) the neural marker neurofilament (NF) as well as for the astroglial and schwann cell markers (D) GFAP and (E) S100, respectively. No Keratin 8 (KER8) staining was observed (F).
(TIF)

Figure S4 **Immunohistochemistry of a typical and an atypical tumor that were generated in GCre;Bmi1^LSL^ transgenic mice shows expression of PCNA (A,B), Ki67 (C, D) and phosphorylated Histone H3 (E, F), respectively.**
(TIF)

Table S1 **IHC analysis of additional markers in Gcre; BmiP^LSL^P induced tumors.**
(DOC)
