# Supplementary figures and images for: GFAP-Cre-Mediated Transgenic Activation of Bmi1 Results in Pituitary Tumors
Source: PLoS One. 2012 May 4;7(5):e35943. doi: 10.1371/journal.pone.0035943 (PMC3344841; doi:10.1371/journal.pone.0035943)

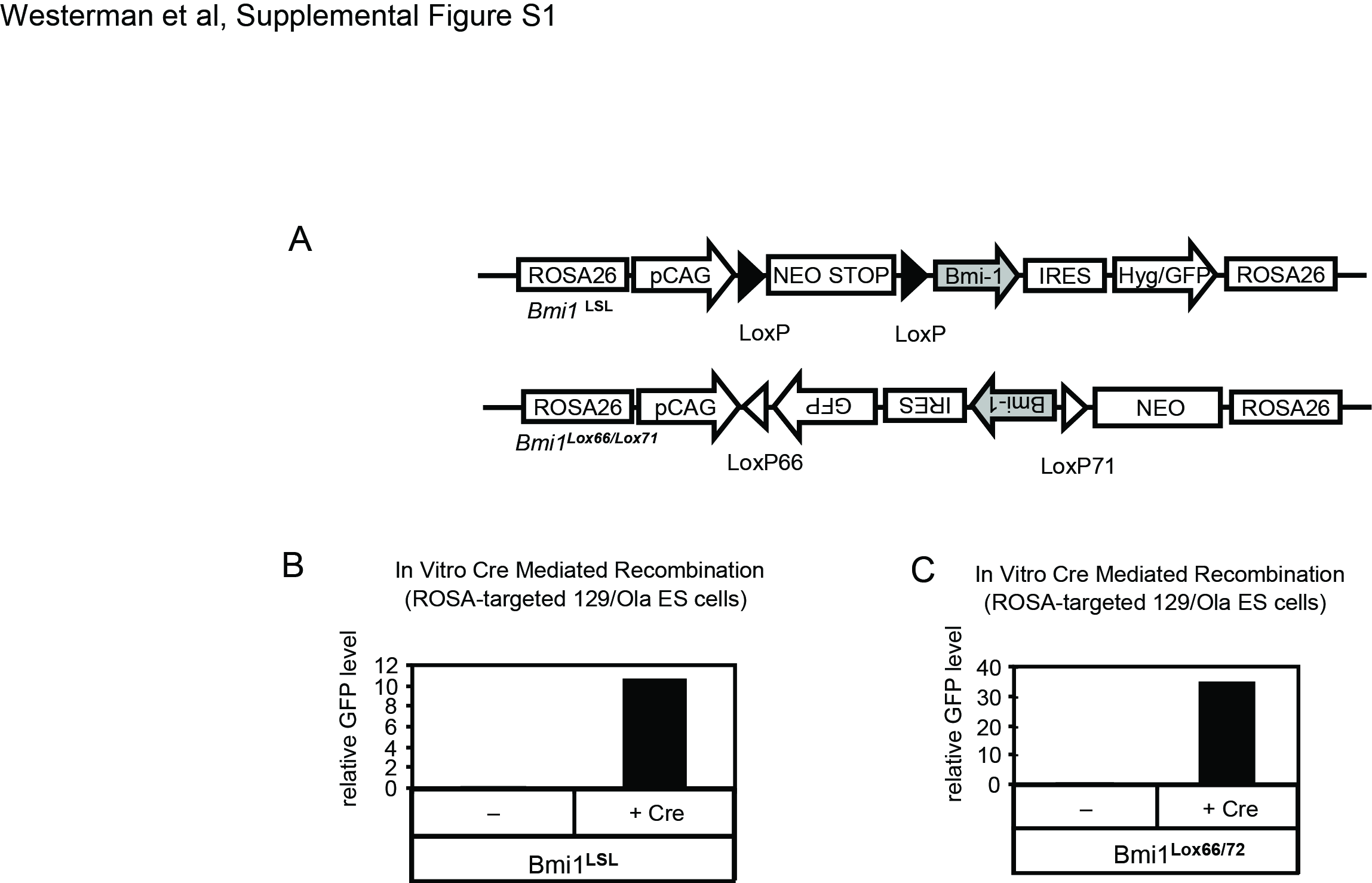

Supplement: Figure S1 — (A) Comparison of the two targeting constructs for conditional over expression of Bmi1. Two LoxP recombination methods are used, the Bmi1LSL construct contains a transcriptional stop sequence that can be removed by LoxP recombination and the Bmi1Lox66/Lox71 construct contains two partially mutated LoxP sites that recombine upon Cre expression resulting reversion of the DNA that is flanked by the LoxP sites which results in the formation of one recombined LoxP site that has a low chance of reversal of the recombination process. ES cells that were targeted with the Bmi1LSL construct (B) or Bmi1LOX66/LOX71 construct (C) show eGFP expression after Cre mediated activation of the transgene. (TIF) [file pone.0035943.s001.tif]

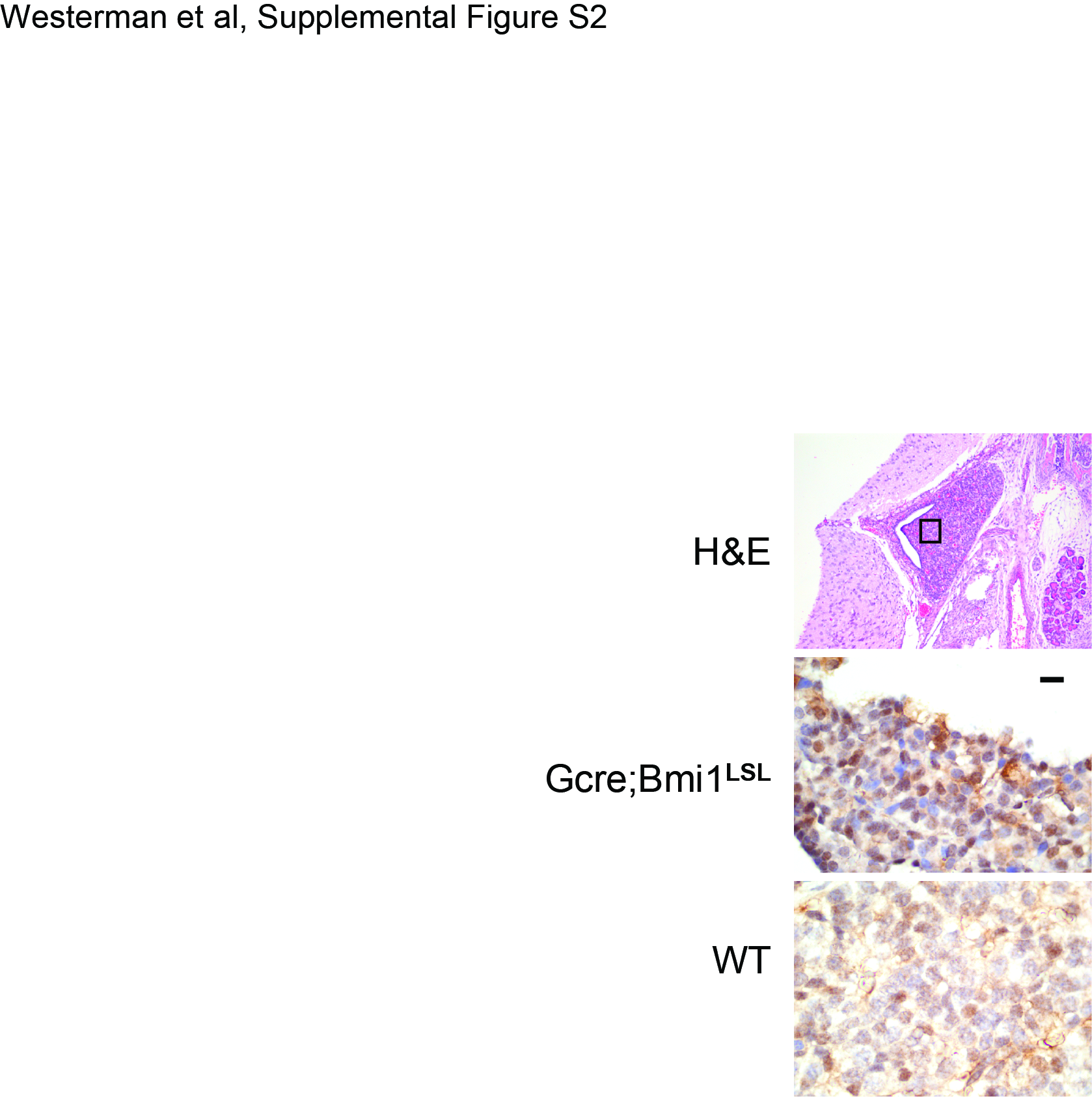

Supplement: Figure S2 — Bmi1 immunohistochemistry of postnatal day 8 pituitary glands (upper panel) shows enhanced expression of Bmi1 in Gcre;Bmi1LSL mice (middle panel) when compared to a wild type controls (lower panel). The Bmi1 antibody was used at a concentration of 1∶50. Bar is 10 µm. (TIF) [file pone.0035943.s002.tif]

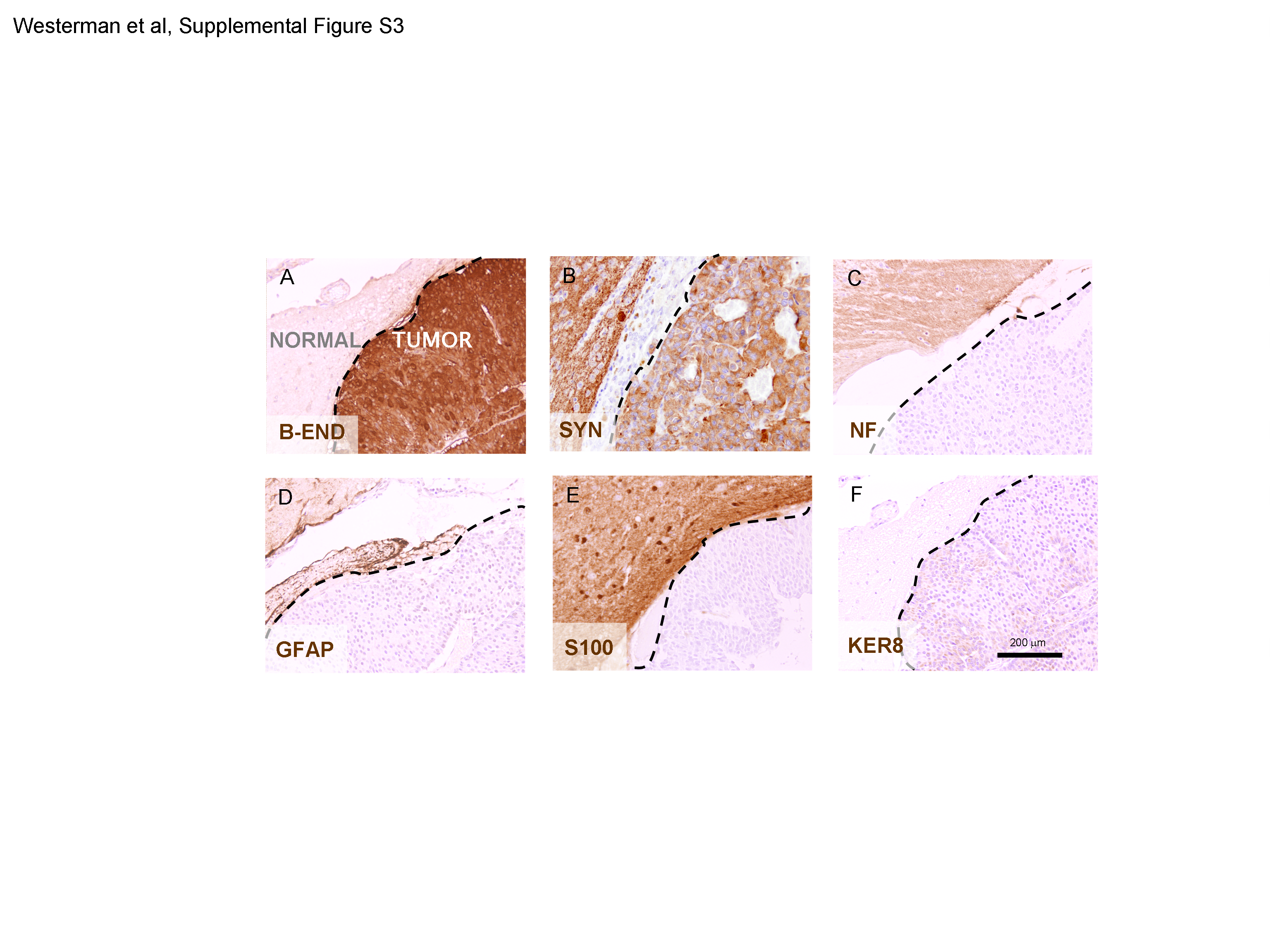

Supplement: Figure S3 — Immunohistochemistry shows that Bmi1 transgenic mice generate pituitary tumors that stain positive for (A) beta-endorphin (B-END) and (B) synaptophysin (SYN). The tumor is negative for (C) the neural marker neurofilament (NF) as well as for the astroglial and schwann cell markers (D) GFAP and (E) S100, respectively. No Keratin 8 (KER8) staining was observed (F). (TIF) [file pone.0035943.s003.tif]

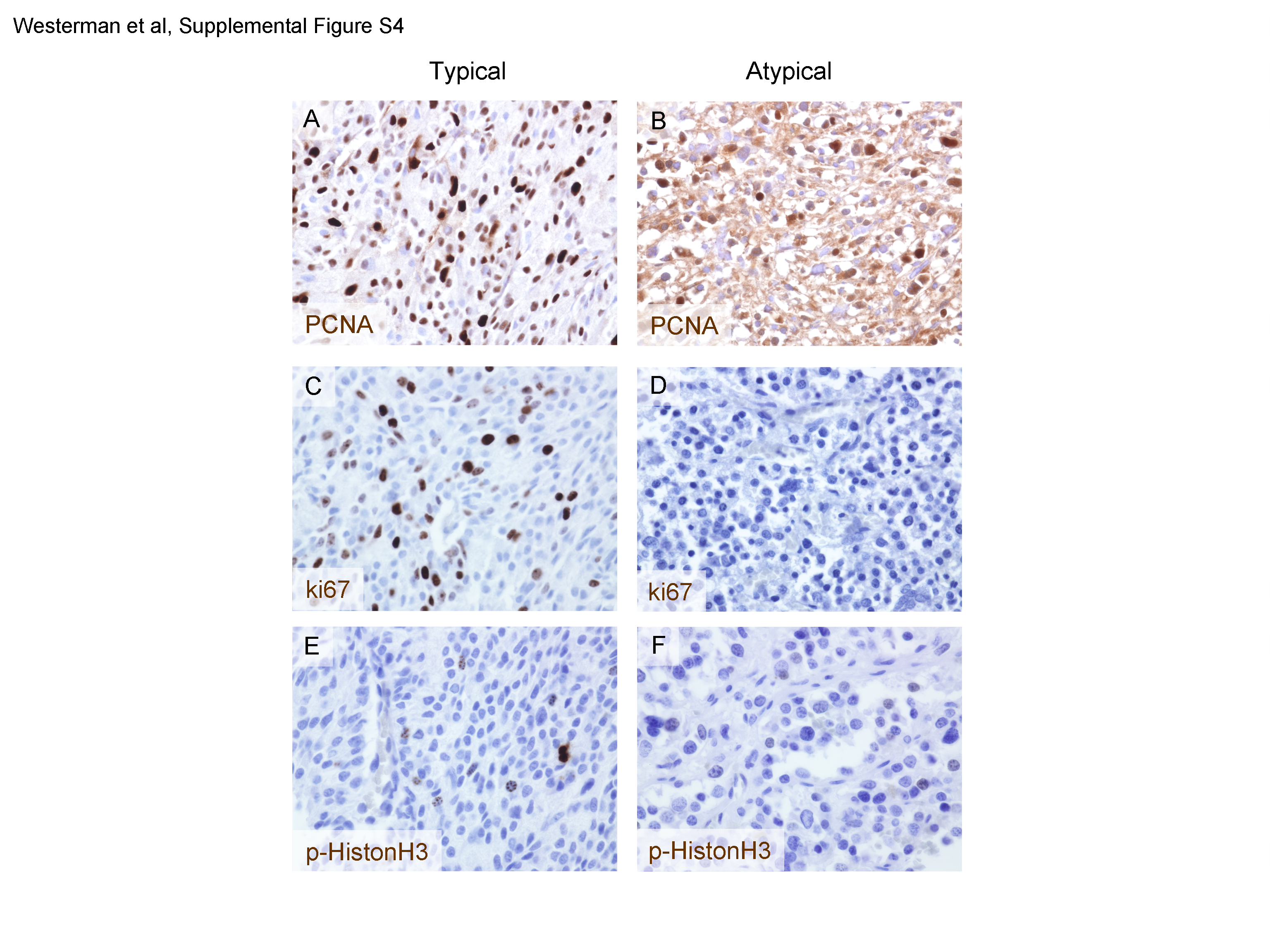

Supplement: Figure S4 — Immunohistochemistry of a typical and an atypical tumor that were generated in GCre;Bmi1LSL transgenic mice shows expression of PCNA (A,B), Ki67 (C, D) and phosphorylated Histone H3 (E, F), respectively. (TIF) [file pone.0035943.s004.tif]
