# Supplementary material for: GFAP-Cre-Mediated Transgenic Activation of Bmi1 Results in Pituitary Tumors
Source: PLoS One. 2012 May 4;7(5):e35943. doi: 10.1371/journal.pone.0035943 (PMC3344841; doi:10.1371/journal.pone.0035943)
Supplement: Table S1 — IHC analysis of additional markers in Gcre; BmiPLSLP induced tumors. (DOC) [file pone.0035943.s005.doc]

| ***Table S1*** | |
| --- | --- |
| ***IHC analysis of additional markers in Gcre; Bmi***P***^LSL^***P ***induced tumors*** | |
|  |  |
| Marker | Typical tumor |
| B-END | **+** |
| SYN | **+** |
| NF | **–** |
| GFAP | **–** |
| S100 | **–** |
| KER8 | **–** |
| CD56/NCAM | **–** |
| CAM5.2 | **–** |
| Chromogranin A | **–** |
